# Design of PV Cells and LEDs Robust to Grid Shadowing Losses in Emission

**DOI:** 10.1021/acsaom.5c00269

**Published:** 2025-09-11

**Authors:** Jasper van Gastel, Pyry Kivisaari, Jani Oksanen, Elias Vlieg, John J. Schermer

**Affiliations:** † 226184 Radboud University, Institute for Molecules and Materials, Applied Materials Science, Heyendaalseweg 135, 6525 AJ Nijmegen, The Netherlands; ‡ 174277 Aalto University, Engineered Nanosystems Group, P.O. Box 13500, FI-00076 Aalto, Finland

**Keywords:** photovoltaics, LEDs, III−V semiconductors, radiative transfer, light management, transfer matrix

## Abstract

In photovoltaics, it is generally assumed that the emission and absorption efficiency is linearly affected by the grid coverage fraction. Typically, the top grid is therefore optimized to allow maximal light exposure with minimal electrical resistance, while the optical properties of the grid are not treated to the same extent. In this work, we provide a numerical study that shows that as a result of the optical properties of the grid, the light extraction efficiency and resulting emission changes nonlinearly with grid coverage, contrary to the standard approximation. If the grid is optically lossy while light is mostly trapped in the diode, the loss in emission is more than linear and therefore larger than expected based on the standard grid shadowing assumption. However, with an optically reflective grid and a good light extraction scheme, the structure obtains a robustness against losses from grid, leading to a meaningful increase in the light extraction efficiency. This is shown using a simple 300 nm GaAs light-emitting diode (LED) structure the emissive properties of which generalize to a thin-film PV cell. Specifically, it is found that depending on the design of the grid and backside mirror, at 10% grid coverage the light extraction efficiency need only be reduced less than 4% absolute. Conversely, in particularly detrimental cases, at 10% grid coverage the light extraction efficiency is reduced by over 35% absolute.

## Graphical abstract

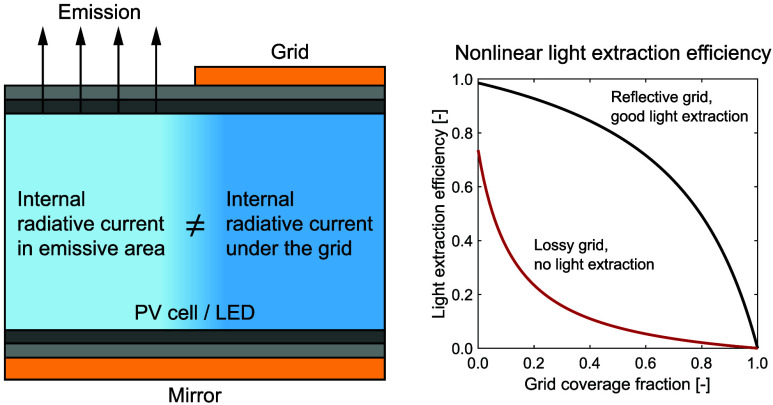

## 1 Introduction

The typical PV cell geometry and common light-emitting diode (LED) geometries have an electrical grid on the front and backside of the device. These are generally optimized to provide low contact resistivity and in high power applications are designed to reduce current spreading issues. On the backside of devices, it is common to consider both the resistivity and reflectivity of the contact, since frequently metallic mirrors are employed to enhance optical performance while simultaneously serving as a low resistivity electrical contact.^1–3^ Contrarily, the reflectivity of the front grid to internal emission is not addressed to the same extent. Since the grid coverage of these devices is generally a small fraction of the total area, the optical properties of the grid are typically assumed not to influence device performance significantly. Optical losses as a result of the grid, generally referred to as shadowing or shading losses, are therefore often assumed to be linear with the grid coverage, for example by correcting the short-circuit current^4^ or photocurrent^5^ for the fraction of the device that is covered by the grid. Recently, however, it was shown by Jeyar et al. for thermophotovoltaic applications in the near-field that metallic grids with widths in the order of the optical wavelength can strongly deteriorate light absorption by TPV cells.^6^ Here, we show that even without invoking microscopic arguments, a nonlinear dependence of the emission on the grid coverage is expected when the internal optical flux of the emitter is different under the grid from the flux at the emissive area.

Nonlinearities in the emitted power as a function of grid coverage are of interest both for the design of LEDs that are optimally emissive and for the design of PV cells that are optimally efficient. In the latter case, this is due to the reciprocity relation between emission and absorption,^7^ which shows that the photovoltaic open circuit voltage of PV cells is enhanced when the external radiative efficiency (and therefore emission via electroluminescence) is large. This effect has been described in detail for GaAs PV cells.^8,9^ As such, even though we limit ourselves to studying the effects of optically lossy grids on emission (as opposed to absorption), the efficiency of solar cells is still affected through its open circuit voltage. For LEDs, it is evident that an increase in light extraction efficiency increases performance. However, while commercial LEDs are focused primarily on enhancing the emitted photocurrent, novel LED devices such as electroluminescent coolers^10–16^ and thermophotonic heat exchangers,^17–25^ require extremely high light extraction efficiencies to operate. Therefore, while for LEDs for lighting a light extraction efficiency boost as a result of grid optimization is welcome but not strictly required, for devices aimed at exchanging heat these considerations are crucial to take into account in the ongoing pursuit of providing operational devices at power densities comparable to e.g., thermoelectric coolers.

In this work we show numerically that the optical properties of the front grid can deteriorate emissive performance far more strongly than linearly as a function of grid coverage when the optical reflectivity of the grids is low and the light is not extracted well from the device (which is often the case in PV cells). Furthermore, we provide guidelines for designing diode structures which are robust to grid coverage, such that the losses in the emission as a result of the grid can be as low as one-third of the grid coverage for reasonable grid coverage fractions. It is shown that good front-side grid for both PV and LED applications should not only be optimized with its electrical properties in mind, but its optical properties should be taken into account as well. More generally, the typical assumption that measured photovoltaic electroluminescence (or LED EQE) can be corrected for active area simply by a linear correction for the grid coverage is not generally correct.

Considering that the typical metrics used in the PV community and LED community are quite different, the results are presented in a way that fits either common practice, or additional information is provided to clarify differences in standard definitions.

## 2 Model Framework

In this work, the TMM-RT model presented in a previous study is employed to determine the optical flux in a biased LED structure.^22,26^ This method uses a radiative transfer approach to determine the steady state flux in the active layer, while the reflectivities, absorptivities and transmissivities of the optical stacks adjacent to the active layer are calculated using the transfer matrix method as presented for example by Centurioni.^27^ The TMM-RT model has shown to produce integrated values of the optical flux accurately when compared to fluctuational electrodymanics calculations, but it is orders of magnitude more numerically efficient and more stable against divergence issues. An abbreviated description of the model is given in this section. The Supporting Information provides the full derivation, including small adaptations to the model as presented in a previous study which was aimed at LEDs coupled to a photodiode for harvesting waste heat, rather than an LED or PV cell emitting to the ambient as is considered here. Comparisons to fluctuational electrodymanics calculations are also provided there, which confirm the validity of the TMM-RT model for this study as well.

In the GaAs active layer, the photon radiance ϕ is emitted and absorbed such that the transport equation in the direction of the unit vector *ẑ* is satisfied, according to

1 $$ẑ\cdot \nabla \phi +\alpha \phi =\frac{\phi_{0}}{\alpha }$$

where α is the absorption coefficient and ϕ_0_ is the equilibrium radiance given by^28^

2 $$\phi_{0}=\frac{n^{2}E^{2}}{\pi^{2}\hbar^{3}c^{2}}\frac{1}{\exp\left(\frac{E-\mu }{\mathrm{kT}}\right)-1}$$

where *n* is the real part of the refractive index of the medium and *E* is the photon energy. We assume the chemical potential μ to be equal to *qV*, where *q* is the elementary charge and *V* is the applied voltage. To more accurately describe the α of GaAs, a convolution with an exponentially decaying tail is applied, such as in several previous studies,^9,22,29,30^ to an absorption coefficient for GaAs based on refractive index data from the Sopra database.^31^ An Urbach energy of 5 meV is used.

In steady state, ϕ does not change after one full round trip through the device, such that at height *z* and angle θ

3 $$\phi \left(z,\theta \right)=\left(1-A_{z,L-z}\right)\phi_{0}+A_{z,L-z}R\left\{\left(1-A_{L,L}\right)\phi_{0}+A_{L,L}R\left[\left(1-A_{L-z,z}\right)\phi_{0}+A_{L-z,z}\phi \left(z,\theta \right)\right]\right\}=G\phi \left(z,\theta \right)+Q\phi_{0}$$

where *L* is the thickness of the active layer. Bold symbols indicate block matrices. Figure 1 schematically depicts this steady state process. Matrix ***A***_*z*_1_,*z*_2__ governs single pass absorption and emission, matrix ***R*** governs reflection and transmission at the interfaces, and matrices ***G*** and ***Q*** are matrices describing all absorption and all emission related terms, respectively, in a complete round trip. The explicit form of these matrices is discussed in the Supporting Information and in previous work.^22,26^ The reflectivity matrix ***R*** can either be parametrized to a set value, or use reflectivities as a function of energy and angle obtained from transfer matrix calculations. The refractive indices of the materials used for transfer matrix calculations in this work are provided in the Supporting Information.

**Figure 1:**
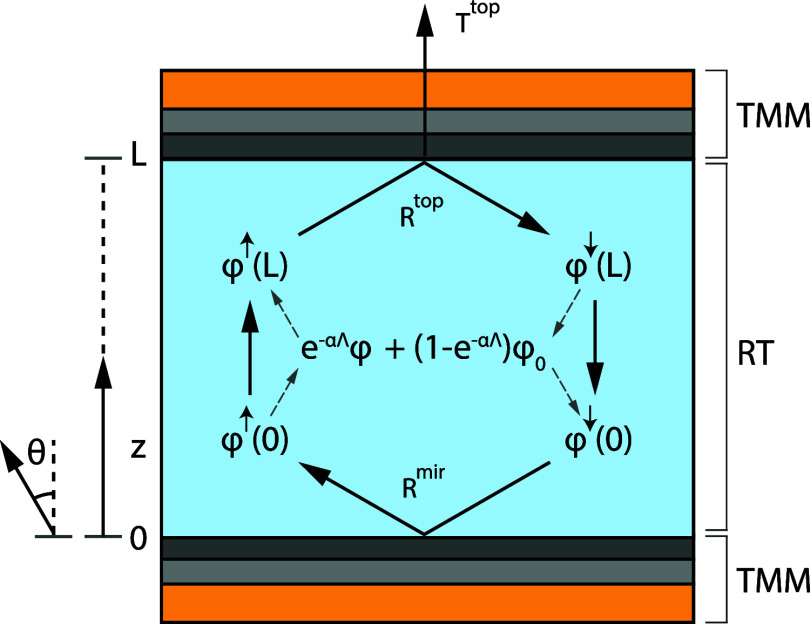
Schematic representation of the steady state equation in the LED or PV structure. RT indicates which part of the structure is considered using radiative transfer, whereas TMM indicates which substructure is considered using the transfer matrix method. Λ = *L*/cos­(θ) represents the total path length through the active layer of the device.

To model scattering surfaces, the surface is parametrized with a haze factor *h*, which is the ratio between specular scattering and Lambertian scattering

4 $$R\left(\theta \right)=hR_{\mathrm{Lamb}}\left(\theta \right)+\left(1-h\right)R_{\mathrm{spec}}\left(\theta \right)$$

The matrices *R*_Lamb_ and *R*_spec_ are discussed in more detail in the Supporting Information. This model neglects the loss in reflectivity as a result of surface plasmon polaritons (SPPs), since the same reflectivity values are used as for planar surfaces, which do not excite SPPs as readily. However, since the use of the haze factor is a crude heuristic to utilize scattering surfaces, this difference in (integrated) reflectivity cannot be straightforwardly estimated for specific materials and as such, is neglected. While this might affect the exact values presented in this work, the general trends are not affected by an offset in reflectivity between planar and scattering mirror structures. To account for SPPs and other potential losses of scattering mirrors, numerical methods such as rigorous coupled wave analyses (RCWA) or finite difference time domain (FDTD) simulations are recommended. The interaction of Lambertian scattering surfaces with SPPs is discussed in more detail for example by Holman et al.^32^

An average reflectance can be determined using a weighted average of matrix ***R*** with angular distribution sin­(θ)­cos­(θ) and energy distribution ϕ_0_(μ = *q*) i.e., at 1 V

5 $$R_{\mathrm{avg}}=\frac{\int_{E}\int_{\theta =0}^{\pi /2}R\phi_{0}\left(\mu =q\right)\cos\theta^{\prime }\sin\theta^{\prime }d\theta^{\prime }dE^{\prime }}{\int_{E}\phi_{0}\left(\mu =q\right)dE^{\prime }\int_{\theta =0}^{\pi /2}\cos\theta^{\prime }\sin\theta^{\prime }d\theta^{\prime }}$$

The average absorptance can be calculated using *A*_avg_ = 1 – *R*_avg_ – *T*_avg_, where *T*_avg_ is the average transmittance calculated the same way as *R*_avg_ using eq 5. Note that *R*_avg_ is barely affected by the choice of voltage, since in the 800 to 900 nm range, where the emission of GaAs is strongest, all reflectance maps used in this study do not change strongly as a function of wavelength.

Equation 3 is solved at the bottom of the active layer *z* = 0 and at the top *z* = *L*. Convergence is reached using an energy range of 1.24 to 1.77 eV (wavelengths of 700 to 1000 nm in steps of 5 nm) and an angular range of 0 to 90° in steps of 0.75°. This yields ϕ­(0, θ) and ϕ­(*L*, θ), from which we determine the net internally generated flux, given by the flux change across a full pass through the LED, according to

6 $$S_{\mathrm{int}}\left(E,\theta \right)=\phi^{↓}\left(0,\theta \right)-\phi^{↓}\left(L,\theta \right)+\phi^{↑}\left(L,\theta \right)-\phi^{↑}\left(0,\theta \right)$$

The change of internal optical flux as a result of interaction with the backside mirror is given by

7 $$S_{\mathrm{mir}}\left(E,\theta \right)=\phi^{↓}\left(0,\theta \right)-\phi^{↑}\left(0,\theta \right)$$

This flux difference is generally lost as a result of absorptive losses. The change of internal optical flux at the topside of the device is given by

8 $$S_{\mathrm{top}}\left(E,\theta \right)=\phi^{↑}\left(L,\theta \right)-\phi^{↓}\left(L,\theta \right)$$

This change is attributed both to emission out of the device and absorption losses to layers between the active layer and the ambient. Using the transfer matrix method for the semiconductor stack at the topside, we find the losses per layer in the stack and the resulting emission to the ambient, defined as *S*_out_(*E*,θ). The internal radiative current density can be calculated directly from *S*_int_(θ) using

9 $$J_{\mathrm{int}}^{\mathrm{rad}}=\frac{q}{2}\int_{E=0}^{\infty }\int_{\theta =0}^{\pi /2}S_{\mathrm{int}}\left(E,\theta \right)\sin\theta^{\prime }\cos\theta^{\prime }d\theta^{\prime }dE^{\prime }$$

where the factor 1/2 is to correct for angular integration. This current describes all radiative current internally generated, which in equilibrium either contributes to photon emission out of the device, or to parasitic optical losses. Reabsorption of readily generated optical flux (i.e., photon recycling) is accounted for inherently by block matrix ***G*** in eq 3. From *J*_int_^rad^, we find the internal radiative saturation current density

10 $$J_{0,\mathrm{int}}^{\mathrm{rad}}=\frac{J_{\mathrm{int}}^{\mathrm{rad}}}{\exp\left(\frac{\mathrm{qV}}{\mathrm{kT}}\right)-1}$$

Note that, since *J*_int_^rad^ scales exponentially with the voltage through its dependence on ϕ_0_, *J*_0,int_^rad^ is a voltage independent property as long as exp­(*qV*/*kT*) ≫ 1.

The radiative current density that contributes to photon emission to the ambient, *J* ^rad^, is given by

11 $$J^{\mathrm{rad}}=\frac{q}{2}\int_{E=0}^{\infty }\int_{\theta =0}^{\pi /2}S_{\mathrm{out}}\left(E,\theta \right)\sin\theta^{\prime }\cos\theta^{\prime }d\theta^{\prime }dE^{\prime }$$

and *J*_0_^rad^ is obtained by dividing *J* ^rad^ by the same exponential term as in eq 10. In general, the internal radiative current can be separated into its constituent loss or emission channels by performing the integration in eq 9 or 11 of its associated component of *S*(*E*, θ). The radiative current is a property common in the PV community, whereas in the LED community it is more common to describe emissive properties in terms of the *B* parameter or spontaneous emission coefficient, which in the current formulation are related by

12 $$B=\frac{J_{0,\mathrm{int}}^{\mathrm{rad}}}{\mathrm{qL}n_{i}^{2}}$$

where *n*_*i*_ is the intrinsic carrier density of GaAs, which we take as 2.23 × 10^12^ m^–3^ at room temperature. All *J*_0,int_^rad^ data presented in the main text is also presented in terms of the *B* parameter in the Supporting Information. The light extraction efficiency is found using

13 $$\eta_{\mathrm{LE}}=\frac{J^{rad}}{J_{int}^{rad}}$$

Since η_LE_ is the fraction between the net radiative currents, it is independent of the internal radiative efficiency η_int_ (which in LED related literature is generally referred to as the IQE).

In previous work, the lateral distance traversed by photons in thin-film GaAs PV cells is estimated to be in the order of several microns.^9^ In this work, the grids are assumed to be wider, such that *J*_int_^rad^ can be calculated separately under the grid and in the area where emission occurs. *J*_int_^rad^ of the full device can then be found from an average between both values of *J*_0,int_^rad^, linearly weighted by the grid coverage fraction *g*_c_. As a consequence, η_LE_ of a full device can be determined by

14 $$\eta_{\mathrm{LE}}=\frac{(1-g_{c})J^{\mathrm{rad}}}{\left(1-g_{c}\right)J_{\mathrm{int},\mathrm{em}}^{\mathrm{rad}}+g_{c}J_{\mathrm{int},\mathrm{grid}}^{\mathrm{rad}}}$$

where subscripts em and grid indicate the emissive area of the device and the area with a grid, respectively. Optical losses, such as to the backside mirror, contact layers or grid, are captured in η_loss_, such that η_LE_ + η_loss_ = 1. This is a notable departure from the PV framework proposed by Steiner et al.^8^ which introduces angle and energy integrated probabilities for a photon to escape, be absorbed and lost parasitically, such that *P*_esc_ + *P*_abs_ + *P*_par_ = 1. Photons that are absorbed with probability *P*_abs_ can thereafter be recycled with probability η_int_, which in turn is defined as the ratio of the radiative recombination rate *U*_rad_ to the total recombination rate *U*_tot_

15 $$\eta_{\mathrm{int}}^{s}=\frac{U_{\mathrm{rad}}}{U_{\mathrm{tot}}}=\frac{U_{\mathrm{rad}}}{U_{\mathrm{rad}}+U_{\mathrm{nr}}}$$

where *U*_nr_ is the nonradiative recombination rate and superscript *s* indicates this is the definition used by Steiner et al. From this, the external radiative efficiency is defined as

16 $$\eta_{\mathrm{ext}}^{s}=\frac{\eta_{\mathrm{int}}^{s}P_{\mathrm{esc}}}{1-\eta_{\mathrm{int}}^{s}P_{\mathrm{abs}}}$$

For LEDs, however, often the light extraction efficiency is defined such that η_LE_η_int_ = η_ext_. Equation 16 shows that the framework by Steiner et al. causes a dependence of η_LE_ on η_int_. To resolve this, the definition for η_int_ used in this work is instead based on the net radiative recombination rate, which in the framework of Steiner et al. can be understood as

17 $$U_{\mathrm{rad},\mathrm{net}}=\left(P_{\mathrm{esc}}+P_{\mathrm{par}}\right)U_{\mathrm{rad}}=\left(1-P_{\mathrm{abs}}\right)U_{\mathrm{rad}}$$

where the subscript net indicates that recycled photons are not included in *U*_rad,net_. We show in the Supporting Information that this allows for a convenient definition of the light extraction efficiency, that does not depend on η_int_ and is therefore constant as a function of voltage

18 $$\eta_{\mathrm{LE}}=\frac{P_{\mathrm{esc}}}{1-P_{\mathrm{abs}}}$$

Furthermore, we show that η_ext_ = η_ext_^*s*^. The formulation by Steiner et al. yields an explicit probability that governs reabsorption, which allows photon recycling to be dealt with in a transparent way. However, the effect of photon recycling is inherently present in the formulation used in this work as well. This is apparent from eq 6, for example, where the difference in the photon radiance between position *z* = 0 and *z* = *L* includes both emission and reabsorption. *J*_int_^rad^ therefore implicitly includes this reabsorption. Despite their apparent difference, both approaches are identical and yield the same results, as shown in the Supporting Information. We prefer the current framework for the purpose of this work, since it allows for the presentation of η_LE_ in a way that generalizes across all values of η_int_ and therefore (voltage) operating points. Since these are generally different between PV cells and LEDs, this framework allows for a presentation of results that is applicable for both LEDs and PV cells. Note that, since η_LE_ is independent of η_int_, the trends in η_LE_ (and *J*_0,int_^rad^) presented in this work are not caused by photon recycling, but rather by a change in photon number (or radiance ϕ) as a result of changes in optical reflectance, absorptance and scattering under the grid and elsewhere. The results presented in this work are therefore general for devices where photon recycling is strong (e.g., GaAs devices), as well as where it is weak (e.g., Si devices), provided they share their geometry with the devices discussed in this work.

For PV cells specifically, the effect of the grid on the open circuit voltage *V*_oc_ can be determined using its relation to η_ext_, according to^7^

19 $$\Delta V_{\mathrm{oc}}=V_{\mathrm{db}}-V_{\mathrm{oc}}=-\frac{\mathrm{kT}}{q}\ln\left(\eta_{\mathrm{ext}}\right)$$

where *V*_db_ is the open circuit voltage in the radiative limit, or detailed-balance voltage. This is the only reported quantity in this work that depends on η_int_, and therefore on the presence of photon recycling.

## 3 Results and Discussion

Before we turn to the effect of the grid coverage on the radiative current and light extraction efficiency, we discuss the structures used in this work. Figure 2 displays reflectivity maps of the optical stack above and below the active layer used throughout this work. The standard structure with a Au mirror (Figure 2e), emitting to air (Figure 2c) and with an optically lossy Pd/Ge/Au alloy grid (Figure 2b) is a simplified rendition of the structure discussed in experimental studies,^33,34^ with *J*_0,int_^rad^ values differing at most 2% from those obtained from the full structure discussed there. We have calculated an effective dielectric function for the Pd/Ge/Au alloy using Bruggemans model with volume fractions of 1/4/10 for Pd/Ge/Au. The other reflectivity maps show optically improved designs additionally discussed in this work. They are chosen to be experimentally feasible rather than theoretically optimal. Au grids have been demonstrated for PV cells without harming contact resistivity,^1,2^ although this cannot be realized ubiquitously, compared to alloyed contacts. MgF_2_/Ag mirrors can be realized using contact points on the back side.^35^ Note that at these contact points, the mirror has no MgF_2_ coating (i.e., it is just Ag). Since the reflectivity of Ag is also high, and the coverage fraction can be as low as 10%, the effect of the contact points on the reflectivity has been neglected.

**Figure 2:**
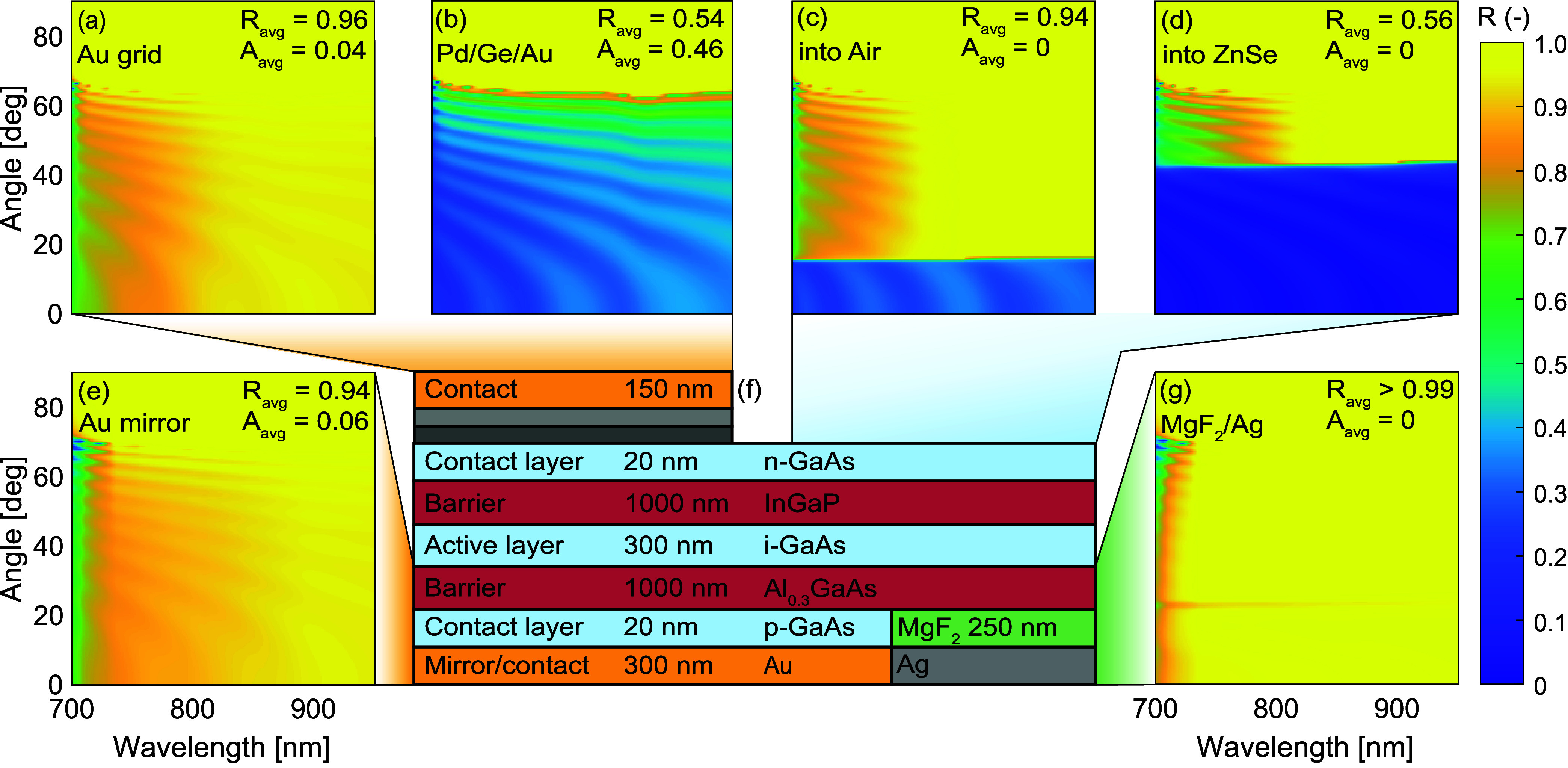
Standard LED structure used throughout this work (f) and TMM reflectivity maps for photons originating from the active layer. The reflectivity of the full material stack above the active layer is shown including Au (a) and Pd/Ge/Au alloy (b) grids. The stack above the active layer without grid is shown in (c, d), representing emission into air and into ZnSe, respectively. TMM reflectivity maps of the material stack below the active layer are shown for a Au mirror (e) and MgF_2_/Ag mirror (g). The average reflectance and absorptance are indicated as well and are calculated using eq 12 in the Supporting Information. Refractive index data used as input for TMM is found in the Supporting Information as well.

The barrier and contact layers are included in the calculations for the reflectivity, which cause interference patterns in the reflectance maps. On energy and angle integrated metrics, this effect is minor. As such, the results presented here are not strongly affected by barrier thickness. For the structures discussed in this work, specifically the n-GaAs contact layer is slightly absorptive and causes the reflectivity to drop below 1 outside the escape cone. Note that strictly speaking, this structure is an LED and not a PV cell, since the top barrier layer is absorptive to solar radiation. However, it is transparent to the emission of the device itself and therefore the results obtained for this device generalize to the emission of PV cells.

An approach taken previously to improve contact resistivity is to anneal Au contacts with Pd and Ge.^34^ While this has proved effective to reduce contact resistivity, Figure 2b shows that this comes at the cost of additional optical losses to the grid, compared to a grid that is only Au (Figure 2a). To improve light outcoupling, two methods are employed in this study. First, encapsulating the emitter in ZnSe improves the outcoupling, since compared to air, its refractive index (*n* ≈ 2.5) is much closer to the refractive index of GaAs (*n* ≈ 3.6). This enhances the outcoupling tremendously, as can be observed by comparing Figure 2c to 2d. Previous studies have experimentally demonstrated the effectiveness of light extraction to ZnSe, reporting near-unity η_ext_.^36,37^ Application of an encapsulating medium is a light extraction method catered to LEDs. The other method is to apply scattering backside mirrors, which also allows the extraction of photon modes that are otherwise trapped internally.^1,38,39^ This method is applicable to both LEDs and PV cells. In addition, usage of a MgF_2_/Ag mirror, which is almost lossless (Figure 2g), is compared to the Au mirror (Figure 2e) used by van der Krabben et al.^33,34^ Note that the difference in reflectivity between Figure 2a and 2e is caused by slight differences between the dielectric function of the n-GaAs and p-GaAs, as well as those of InGaP and Al_0.3_GaAs.

Using the structure and reflectivity maps from Figure 2, we present in Figure 3 the *J*_0,int_^rad^ values as a function of mirror and grid reflectivity shown for several device architectures and emission management strategies. Figure 3a reveals that employing any emission management scheme considered in this work (mirror texturing, ZnSe encapsulation or both) causes a large increase in *J*_0,int_^rad^. Since more light is extracted in those cases, the reabsorption of photons in the active layer is diminished, resulting in a larger net radiative current. However, the same holds for a mirror that is lossy, as *J*_0,int_^rad^ is larger at smaller values of *R*_b_. It is irrelevant how the photons escape the active layer (i.e., either by being emitted or by being lost) for *J*_0,int_^rad^. As such, *J*_0,int_^rad^ is not straightforwardly a metric to judge the quality of a PV cell or LED. Rather, it yields insight in the efficacy of photon generation in the active layer specifically, either through emission or though loss. Figure 3b shows the same metrics as Figure 3a, now for devices that are fully covered (*g*_c_ = 1), to assess the effect of lossy grids on *J*_0,int_^rad^. Optically lossy grids, such as the Pd/Ge/Au grid, cause a large increase in *J*_0,int_^rad^, similar to lossy mirrors.

**Figure 3:**
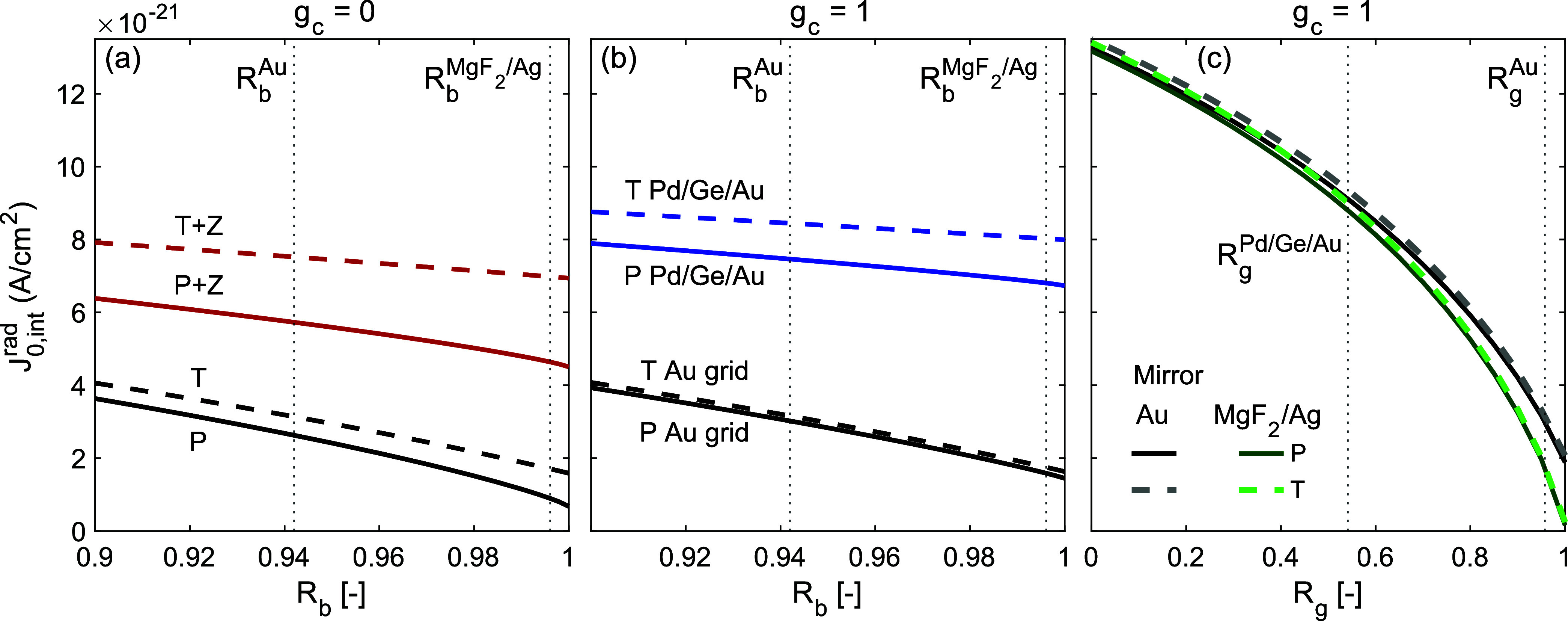
Trends in *J*_0,int_^rad^ as a function of backside reflectance and grid reflectance for different device architectures. (a) *J*_0,int_^rad^ for LEDs with planar (P) and textured (T) mirrors with a haze factor of *h* = 0.5 emitting to air or into ZnSe (Z). (b) *J*_0,int_^rad^ for LEDs emitting into a reflective grid (Au) and lossy grid (Pd/Ge/Au alloy). (c) *J*_0,int_^rad^ for LEDs with different mirror architectures, with planar and textured mirrors. The average reflectances of different grid and mirror structures from Figure 2a are indicated with vertical dotted lines. The same data plotted as *B* parameter values is shown in the Supporting Information.

Preluding to the analyses of the nonlinear relation between η_LE_ and *g*_c_, the decrease in η_LE_ as a function of grid coverage becomes superlinear when *J*_int,grid_^rad^ > *J*_int,em_^rad^, e.g., for the cases without ZnSe encapsulation in Figure 3a combined with the Pd/Ge/Au grid in Figure 3b, since the total *J*_int_^rad^ is the weighted sum of *J*_int_^rad^ under the grid and at the emissive area, as in eq 14. Conversely, when *J*_int,grid_^rad^ < *J*_int,em_^rad^, the decrease in η_LE_ as a function of grid coverage is less than linear, e.g., for the cases with encapsulation in Figure 3a combined with the Au grid in Figure 3b. In that instance, the device becomes partially robust to losses as a result of the grid. The sensitivity of *R*_g_ on *J*_int_^rad^ is highlighted in Figure 3c. The range in which *J*_0,int_^rad^ varies there is larger than the range in which *J*_0,int_^rad^ varies for the structures considered in Figure 3a,b, which emphasizes the need to account for the grid reflectivity to prevent unnecessary optical losses. Note that *R*_g_ in Figure 3c is parametrized to a constant value for all angles and energies and as such, there is next to no difference between planar and textured mirrors. This is a departure from the trends presented in Figure 3b, where *R*_g_ depends on propagation angle and wavelength. Since the reflectivity of the Au grid is nearly constant as a function of angle and wavelength, the approximation is negligible in that case. For the Pd/Ge/Au grid, there is a clear escape cone-like structure in the reflectivity map as shown in Figure 2b, i.e., there is a strong dependence on angle. This causes a small discrepancy between the parametrized *R*_g_ in Figure 3c and the wavelength and angle dependent *R*_g_ in Figure 3b.

Expanding on the above, Figure 4 combines *J*_0,int_^rad^ from Figure 3a,3b with *g*_c_ = 0.1, separated into the different channels through which photons are lost or emitted from the device. In the structures considered here, these channels are emission out of the device (*J*_0_^rad^), losses to the mirror with parametrized, angle and energy independent reflectivity, to the n-GaAs contact layer on the top side of the structure and to the grid. With this setup, we aim to contrast devices with an optically lossy grid and poor light extraction schemes (top row) with devices with a reflective grid and good light extraction schemes (bottom row). The *J*_0,int_^rad^ of devices presented on the top row therefore emit to air instead of ZnSe and have a Pd/Ge/Au grid, which highlights that massive losses can be attributed to the grid. On the bottom row, light extraction to ZnSe is considered and the grids are Au, in which case the losses to the grid become negligible.

**Figure 4:**
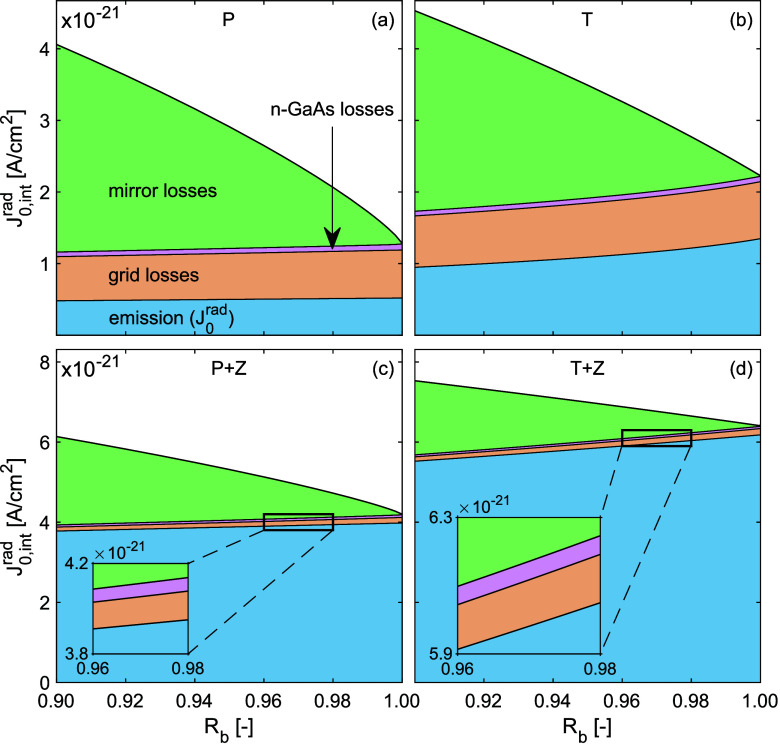
Trends in *J*_0,int_^rad^ as a function of backside reflectance broken down into separate loss and emission channels for devices with different light extraction schemes and a grid coverage of *g*_c_ = 0.1. (a) with a planar backside mirror and a lossy PD/Ge/Au grid (b) with a textured mirror with *h* = 0.5 and a lossy PD/Ge/Au grid (c) with a planar mirror, ZnSe encapsulation and a Au grid, and (d) with a textured mirror with *h* = 0.5, ZnSe encapsulation and a Au grid. The insets on the bottom row highlight the grid and n-GaAs loss channels.

Figure 4a indicates that, in the absence of a light extraction scheme (i.e., planar structure), mirror losses contribute most to *J*_0,int_^rad^ at reflectivities up to 98%. Only at near-unity reflectivities does its contribution become small compared to the other channels. Therefore, one of the key design principles for efficient (thin-film) LEDs and PV cells is to ensure the mirror has near unity reflectivity. This has been shown extensively in previous studies.^9,39^ Perhaps more strikingly, even at 10% grid coverage, the optical losses to the grid are larger than the emission out of the device. This is the case since the angular dependence of the optical flux follows sin­(θ) cos­(θ), so that most flux is directed at an angle of 45°, which is markedly larger than the escape cone from the device of about 16°. In contrast, the grid is optically lossy under all angles of incidence. Integrated over all angles, it therefore accounts for a larger fraction of *J*_0,int_^rad^.

Comparing the structures with planar and textured mirrors without ZnSe encapsulation (Figure 4a,b, respectively), we find that, although *J*_0,int_^rad^ is slightly larger with a textured mirror, the relative component of losses to the mirror is smaller. Therefore, the increase in *J*_0,int_^rad^ is caused by a strongly enhanced emission from the device. This emission enhancement is due to the scattering of photons that would otherwise be confined to modes outside of the escape cone of the device. As a result of scattering, the emission out of the device is larger than the grid losses. When applying ZnSe encapsulation and replacing Pd/Ge/Au grids with Au as on the bottom row of Figure 4, the emission becomes the dominant contributor to *J*_0,int_^rad^, while the losses to the grid become negligibly small, similar to the losses to the n-GaAs contact layer. While the escape cone from GaAs to air is about 16°, it is about 45° for emission from GaAs to ZnSe. This results in a smaller relative increase of the emission from the device as a result of a textured mirror (Figure 4c,d, respectively) compared to the case without ZnSe encapsulation. However, since the magnitude of the emission is far larger into ZnSe, the absolute increase in emission due to a textured mirror is still larger with ZnSe encapsulation. Comparing Figure 4a to Figure 4d, which exhibit the largest difference in outcoupling, reveals that not only the fraction of *J*_0,int_^rad^ lost to the mirror is smaller when light outcoupling is good, but the absolute internal radiative current lost to the mirror becomes smaller as well. This happens in spite of a more than 2-fold increase in *J*_0,int_^rad^. It shows that the total amount of photons lost to the mirror decreases, regardless of the increase in *J*_0,int_^rad^, when emission becomes the dominant channel through which photons escape the active layer. In section 6 of the Supporting Information, the same structure is discussed with a 2000 nm active layer, which is more typical for III–V PV cells. While *J*_0,int_^rad^ is about twice as large, the trends as a function of *R*_b_ are extremely similar. It is notable, however, that the impact of the light extraction scheme is lower if the active layer is thicker. Similarly, a structure is discussed in the same section of the Supporting Information in which the contact layers are 200 nm in thickness, instead of 20 nm. It is shown that even for thick contact layers, the losses are small compared to losses to an absorptive Pd/Ge/Au grid. However, thick contact layers contribute strongly to the total optical losses when the mirror and the grid are highly reflective. It exemplifies the need for either very thin, or transparent contact layers (e.g., Al_0.3_GaAs), to prevent additional optical losses in those cases.

It follows from eq 14 that the light extraction efficiency η_LE_ does not depend linearly on the coverage of the grid. Crucially, from Figure 3 it can be observed that *J*_0,int_^rad^ varies in a similar range both under the grid and elsewhere. Consequently, *J*_0,int_^rad^ under the grid can be either larger or smaller than *J*_0,int_^rad^ at the emissive area of the device (as opposed to one of them always being larger than the other). As a result, in a structure that has excellent light extraction and a reflective grid, η_LE_ becomes robust to shadowing losses resulting from the grid, such as in Figure 5(a),(c) when ZnSe encapsulation is used (for both planar and textured mirrors). In those cases, η_LE_ decreases slower than linear as a function of *g*_c_. In the most extreme case, with a textured MgF_2_/Ag mirror, Au grid and ZnSe encapsulation, a 10% absolute reduction in η_LE_ is incurred at a grid coverage as high as 31% as shown in Figure 5(c). Contrarily, if light is not extracted efficiently while the grid is lossy, η_LE_ decreases faster than linear as a function of *g*_c_. For example, for a device with a planar MgF_2_/Ag mirror, Pd/Ge/Au grid and no encapsulation, such as in Figure 5(d), a 10% absolute reduction in η_LE_ is incurred already at a grid coverage of 2% and a 50% absolute η_LE_ reduction in η_LE_ is incurred at 20% grid coverage. Put differently, if PV cell or LED grids are designed optimally, at 10% grid coverage η_LE_ is only reduced by 3.5% absolute. Conversely, in particularly detrimental cases, at 10% grid coverage η_LE_ is reduced by 35.8% absolute.

**Figure 5:**
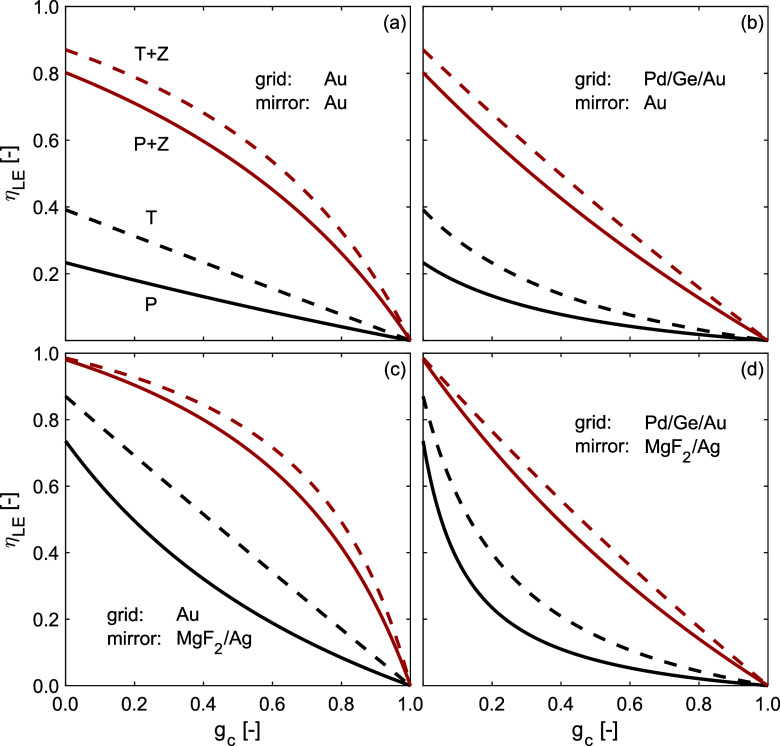
η_LE_ as a function of grid coverage for different device architectures and light extraction schemes. (a) With Au grid and mirror, (b) with Pd/Ge/Au alloy grid and Au mirror, (c) with Au grid and MgF_2_/Ag mirror and (d) with Pd/Ge/Au alloy grid and MgF_2_/Ag mirror. All trends are displayed for devices with planar (P) and textured (T) mirrors with a haze factor of *h* = 0.5 emitting to air or into ZnSe (Z). The same data plotted as *B* parameter is shown in the Supporting Information.

For PV cells specifically, another way to gauge this phenomenon is through Δ*V*_oc_, which is the *V*_oc_ penalty with respect to the radiative limit. The trends in Δ*V*_oc_ are plotted in the Supporting Information, based on the data of Figure 5. Assuming η_int_ = 1 and only looking at the devices emitting to air, which is typical for PV cells, Δ*V*_oc_ penalty is about 4 mV smaller using a Au grid instead of a Pd/Ge/Au grid, regardless of texturization, when a lossy Au mirror is used. When a MgF_2_/Ag mirror is employed, Δ*V*_oc_ is 12 mV smaller when a Au grid is used, compared to a Pd/Ge/Au grid, when the mirror is planar, and 8 mV smaller when the mirror is textured. Especially in the latter cases, this *V*_oc_ penalty can have a significant effect on the power conversion efficiency of PV cells, which scales linearly with the *V*_oc_.

For experimental verification of nonlinear optical losses to the grid, two structures from the same growth run could be processed with different grids (e.g., Au and Pd/Ge/Au) and characterized. Either their ratio of η_LE_ can be compared, which is the same as the ratio of η_ext_ if η_int_ is the same in both devices. If the devices are from the same growth run, η_int_ should not differ much between them, as it is largely based on the quality and doping of the active layer and interfaces, which should be the same in that case. η_ext_ can be measured directly using a spectrometer and an integrating sphere. Alternatively, one can perform PV characterization and compare the *V*_oc_. This is less accurate, however, since it depends logarithmically on η_ext_, which reduces sensitivity, and moreover, also depends on *V*_db_. This quantity can differ as a result of the grid as well, since the grid can affect *J*_0_^rad^, thereby convoluting the direct effect on the *V*_oc_.

The aforementioned discussion indicates that a good front-side grid for both PV and LED applications should not only be optimized with its electrical properties in mind, but its optical properties should be taken into account as well. More generally, the typical assumption that measured photovoltaic electroluminescence or LED EQE can be corrected for active area simply by dividing it by 1 – *g*_c_ is not generally correct. By aiming to reduce optical losses of the grid (and other layers), PV cells and LEDs can be designed that are partially robust to losses in emission as a result of the grid. Predictions for optically improved designs can be made by simulations such as presented in this work. However, optimization of optical properties through grid design should not come at the cost of electrical performance, for example due to an increase in series resistance, a decrease in conductivity or suboptimal current spreading. Rather, optical simulations of the grid and cell as a whole should be performed in conjunction with experimental determination of electrical properties, such that a balance can be found between both that maximizes performance.

## 4 Conclusions

This study presents a comparison of the internal radiative current, radiative current that leads to emission and light extraction efficiency for several typical LED or PV cell structures. It is shown that the when the radiative current under the grid and under the emissive area of the device differ, the light extraction efficiency changes nonlinearly as a function of grid coverage. This study therefore highlights that calculating the grid shadowing effect solely based on the fraction of the device covered by grid lines is too simplistic. Since the range in which the radiative current varies is similar under the grid and at the emissive area, proper design of PV and LED structures yields devices in which the light extraction efficiency is not affected as strongly by the grid coverage at moderate coverage fractions. For a realistic LED design at 10% grid coverage, η_LE_ is only reduced by 3.5% absolute. Contrarily, lossy grids combined with inefficient light extraction methods, cause a reduction in the light extraction efficiency far larger than the coverage fraction. This can cause a reduction in η_LE_ of 35.8% absolute at 10% grid coverage in feasible LED structures. This highlights the need to consider both the optical and electrical properties of the grids on PV and LED devices.

## Supplementary Material



## Notes

Derivation of TMM-RT model; refractive index data used; supporting plots for *J*_0,int_^rad^ as *B* parameter; comparisons to fluctuational electrodynamics simulations; derivation of equivalence of presented framework and that of Steiner et al.;^8^ analyses of structures with 2000 nm active layer and 200 nm contact layers (PDF)
