# Supplementary material for: Design of PV Cells and LEDs Robust to Grid Shadowing Losses in Emission
Source: ACS Appl Opt Mater. 2025 Sep 11;3(9):2153–62. doi: 10.1021/acsaom.5c00269 (PMC12481568; doi:10.1021/acsaom.5c00269)
Supplement: Supplementary file 1 [file ot5c00269_si_001.pdf]

Supporting Information for: Design of PV cells and LEDs robust to grid shadowing losses in emission

Jasper van Gastel*[1], Pyry Kivisaari[2], Jani Oksanen[2], Elias Vlieg[1] and John J. Schermer[1]

[1]*Radboud University, Institute for Molecules and Materials, Applied Materials Science - Heyendaalseweg 135, 6525 AJ, Nijmegen, The Netherlands*
[2]*Aalto University, Engineered Nanosystems Group, P.O. Box 13500, FI-00076 Aalto, Finland*

1 Derivation of coupled radiative transfer - transfer matrix model

In this work, we use a radiative transfer model in the active layer of the LED or PV device, while using transfer matrix calculations to calculate the reflectivity and transmissivity of the semiconductor stacks above and below the active layer. Here we provide the full description of the TMM-RT model as employed in the main text, including (minor) deviations to the model presented in our previous work.[1]
In the active layers, the photon radiance ϕ is emitted and absorbed such that the transport equation in the direction of the unit vector $\hat{z}$ is satisfied, according to

$$\hat{z} \cdot \nabla\phi + \alpha\phi = \frac{\phi_0}{\alpha}, \tag{1}$$

where α is the absorption coefficient and ϕ_0 is the equilibrium radiance given by[2]

$$\phi_0 = \frac{n^2E^2}{\pi^2\hbar^3c^2}\frac{1}{\exp\left(\frac{E-\mu}{kT}\right)-1}, \tag{2}$$

where n is the real part of the refractive index of the medium and E is the photon energy. We assume the chemical potential μ to be equal to qV. ϕ_0/α is the spontaneous recombination rate obtained by Würfel.[3]
From equation 1 we find the single pass absorption and emission in the active layer

$$\phi(L,\theta) = \phi(0,\theta)e^{-\alpha L} + \left(1-e^{-\alpha L}\right)\phi_0, \tag{3}$$

where $L = t/cos(\theta)$ is the path length in which t is the thickness of the active layer and θ is the angle of propagation with respect to the normal of the device. The first term in equation 3 describes

*Corresponding author: jasper.vangastel@ru.nl

the absorption, while the second term accounts for internal emission. Reflection and transmission inside the device are accounted for by multiplication with the respective reflection or transmission coefficient. In steady state, ϕ does not change after one full round trip through the device, such that

$$\begin{aligned} \phi(z,\theta) = (\mathbb{1} - \boldsymbol{A}_{z,L-z})\phi_0 + \boldsymbol{A}_{z,L-z}\boldsymbol{R}\{(\mathbb{1} - \boldsymbol{A}_{L,L})\phi_0 \\ + \boldsymbol{A}_{L,L}\boldsymbol{R}[(\mathbb{1} - \boldsymbol{A}_{L-z,z})\phi_0 + \boldsymbol{A}_{L-z,z}\phi(z,\theta)]\} = \boldsymbol{G}\phi(z,\theta) + \boldsymbol{Q}\phi_0, \end{aligned} \tag{4}$$

where bold symbols are used to indicate block matrices. Matrix $\boldsymbol{A}_{z_1,z_2}$ governs single pass absorption and emission, matrix $\boldsymbol{R}$ governs reflection and transmission at the interfaces, and matrices $\boldsymbol{G}$ and $\boldsymbol{Q}$ are matrices describing respectively all absorption related terms and all emission related terms in a complete round trip. The explicit form of these matrices is discussed below. Figure S1 shows the steady state process which yields equation 4. The height coordinate z ranges from 0 to L for each angle θ. ϕ is described as a composite vector containing upwards and downwards propagating radiance vectors so that $\phi(z,\theta) = (\phi^{\uparrow}(z,\theta), \phi^{\downarrow}(z,\theta))$.
The block matrices $\boldsymbol{A}_{z_1,z_2}$ governing absorption and emission are given by

$$\boldsymbol{A}_{z_1,z_2} = \begin{pmatrix} a(z_1) & 0 \\ 0 & a(z_2) \end{pmatrix}, \tag{5}$$

where $a(z) = \mathrm{diag}(\exp(-\alpha z/\cos(\theta)))$ is a diagonal matrix containing the elements for all θ. $z/\cos(\theta)$ is the pathlength through the active layer of the device at angle θ from 0 to height z. In $\boldsymbol{A}_{z_1,z_2}$, index z_1 is associated with flux propagating toward the top side of the device (i.e. from $z = 0$ to $z > 0$) and z_2 is associated with flux propagating toward the mirror. Block matrix $\boldsymbol{R}$ that governs reflection and transmission is given by

$$\boldsymbol{R} = \begin{pmatrix} 0 & R^{mir}(\theta) \\ R^{top}(\theta) & 0 \end{pmatrix}. \tag{6}$$

It accounts for reflection at the interfaces of the device and transmission out of the device. Each sub-matrix for reflection $R(\theta)$ has a similar structure and is calculated using the TMM such as described in.[4] Matrices $R(\theta)$ in block matrix $\boldsymbol{R}$ are of size θxθ so that the flux from any angle of incidence can be mapped to flux in any angle of reflection. It accounts for reflection at the interfaces of the device and transmission between the LED and PD sides. We consider surfaces that scatter specularly, as well as according to the Lambertian distribution. For planar surfaces, $R(\theta)$ are diagonal matrices (i.e. $\theta_{in} = \theta_{out}$). For scattering surfaces, we use the Lambertian distribution given by

$$\phi_{out}(\theta_{out}) = \frac{\cos\theta_{out}\sin\theta_{out}}{\int_{\theta=0}^{\pi/2}\cos\theta'\sin\theta' d\theta'}\int_{\theta=0}^{\pi/2}\phi_{in}d\theta' = \frac{1}{2}\cos\theta_{out}\sin\theta_{out}\int_{\theta=0}^{\pi/2}\phi_{in}d\theta', \tag{7}$$

where the cosine term describes Lambertian scattering and the sine term accounts for the azimuthal angle. The integral in the numerator accounts for normalization. In the present context, this gives the matrix form

$$R_{row}(\theta) = \frac{R'(\theta)}{2}\cos(\theta_{out})\sin(\theta_{out}), \tag{8}$$

where $R'(\theta)$ is the reflection element found using the TMM and is different for each row. θ_{out} represents the outgoing angle. Experimentally obtained rough surfaces generally do not generally

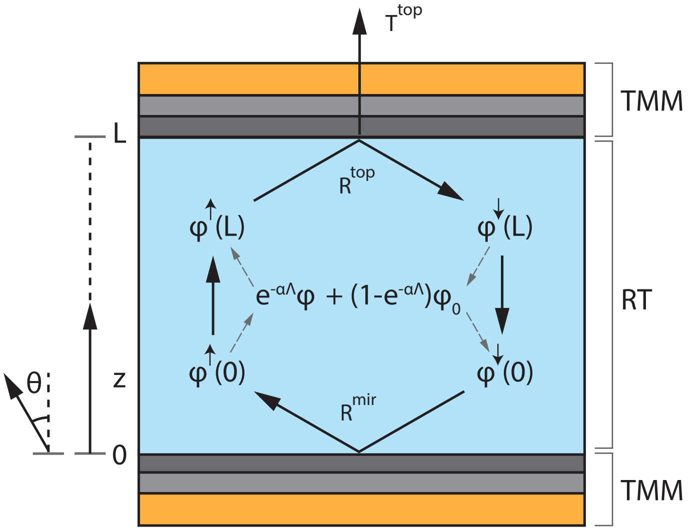

Figure S1: Schematic representation of the steady state equation in the LED or PV structure. RT indicates that part of the structure is considered using radiative transfer, whereas TMM indicates that sub-structure is considered using the transfer matrix method.

scatter light according to the Lambertian distribution. We therefore parameterize the surfaces with a haze factor h, which is the ratio between specular scattering and Lambertian scattering

$$R(\theta) = hR_{Lamb}(\theta) + (1-h)R_{spec}(\theta). \tag{9}$$

The explicit forms of $\boldsymbol{G}$ and $\boldsymbol{Q}$ are obtained by rewriting equation 4. $\boldsymbol{G}$ captures all terms corresponding to absorption in the device, given by

$$\boldsymbol{G} = \boldsymbol{A}_{L-z,z}\boldsymbol{R}\boldsymbol{A}_{L,L}\boldsymbol{R}\boldsymbol{A}_{z,L-z}, \tag{10}$$

while $\boldsymbol{Q}$ accounts for the internal emission and is given by

$$\boldsymbol{Q} = \mathbb{1} - \boldsymbol{A}_{L-z,z} + \boldsymbol{A}_{L-z,z}\boldsymbol{R}(\mathbb{1} - \boldsymbol{A}_{L,L} + \boldsymbol{A}_{L,L}\boldsymbol{R}(\mathbb{1} - \boldsymbol{A}_{z,L-z})). \tag{11}$$

Equation 4 is solved at $z = 0$ and $z = L$ for each energy E and angle θ of interest. This yields $\phi(0, \theta)$ and $\phi(L, \theta)$, from which the net emitted flux is found, which is discussed in the main text.
For comparison of $\boldsymbol{R}$ to a parametrized, angle and wavelength independent reflectivity, an average reflectivity can be determined using a weighted average of matrix $\boldsymbol{R}$ with angular distribution $\sin(\theta)\cos(\theta)$ and and energy distribution of $\phi_0(\mu = q)$ i.e. at 1 V

$$R_{avg} = \frac{\int_E \int_{\theta=0}^{\pi/2} \boldsymbol{R}\phi_0(\mu = q)\cos\theta' \sin\theta' d\theta' dE'}{\int_E \phi_0(\mu = q)dE' \int_{\theta=0}^{\pi/2} \cos\theta' \sin\theta' d\theta'}. \tag{12}$$

The average absorptance A_{avg} can be calculated using $1 - R_{avg} - T_{avg}$, where the latter is the average transmissivity calculated using equation 12.

2 Refractive index data

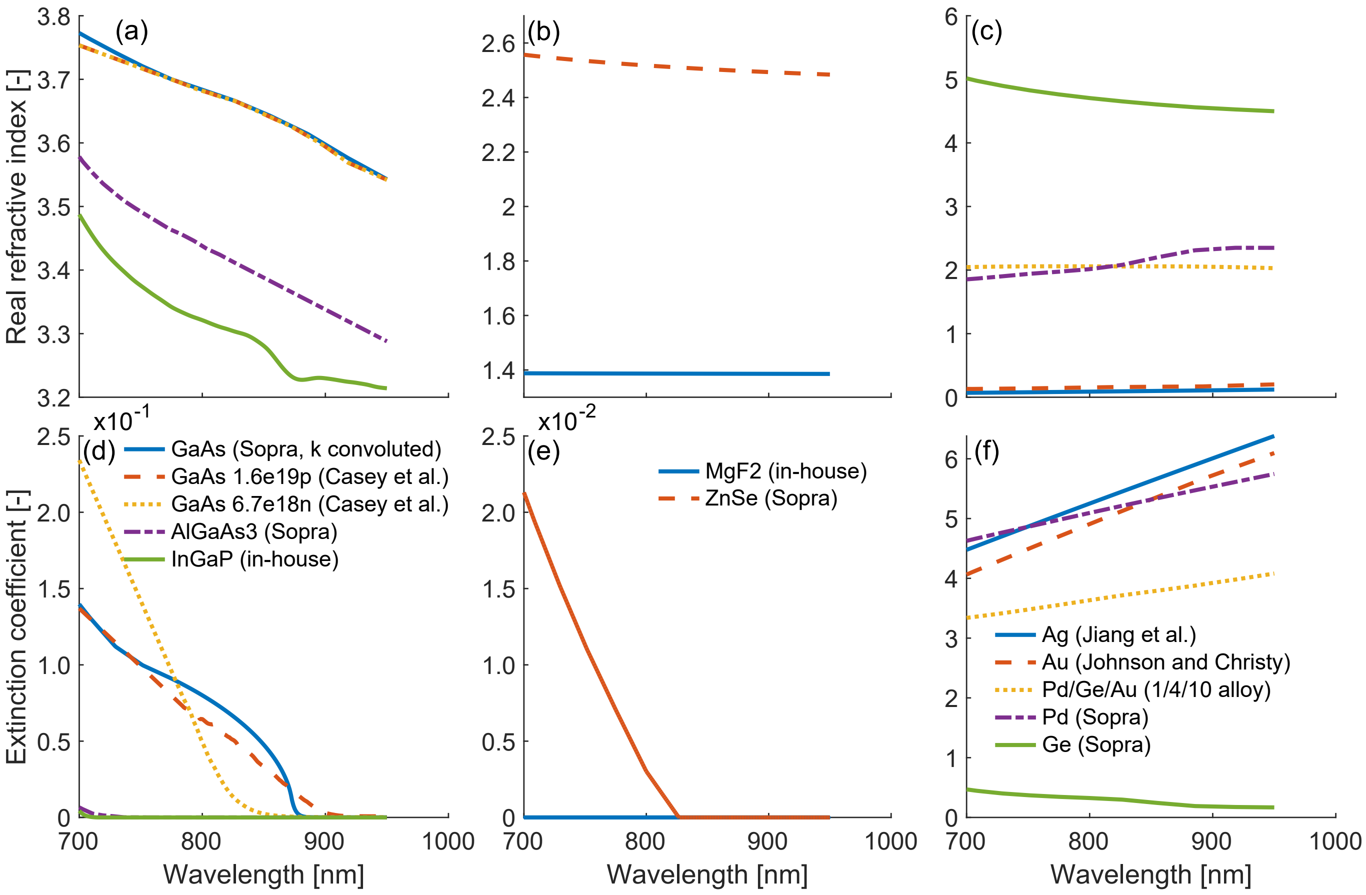

Figure S2: Refractive index data used throughout this work. Legends apply per column and are given on the second column for clarity. (a) Real part of the refractive index of semiconductor materials used in the epitaxial stack of the simulated structures in the main text ((d) shows the imaginary part). Note that the intrinsic GaAs data from Sopra[5] that is used for the active layer has an Urbach tail applied by the authors via convolution with an Urbach energy of 5 meV. (b) Real part of the refractive index of dielectric materials used in the simulated structures ((e) shows the imaginary part). (c) Real part of the refractive index of metals and other materials used in metal alloys present in the simulated structures ((f) shows the imaginary part). The Pd/Ge/Au alloy refractive index has been calculated using Bruggemans model with volume ratios of 1/4/10 respectively. Refractive index data for Ag is sourced from Jiang et al.[6] For Au, it is sourced from Johnson and Christy.[7] For doped GaAs, it is sourced from Casey et al.[8] Refractive index data for intrinsic GaAs and all other materials are sourced from the Sopra database.[5]

3 Spontaneous emission coefficient

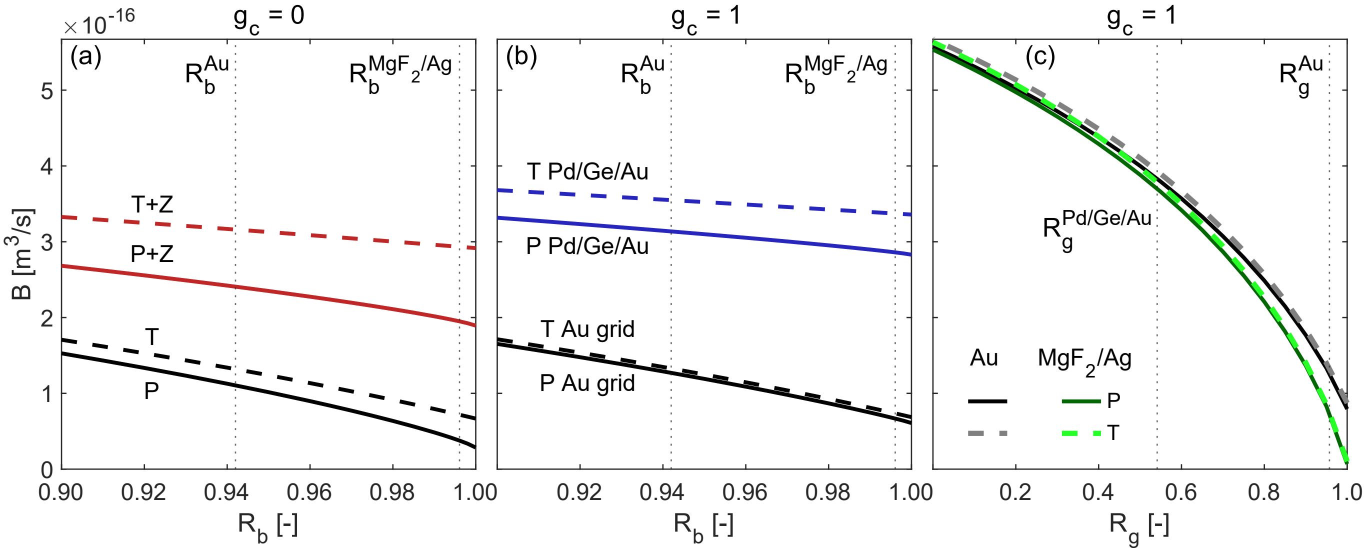

Figure S3: Trends in spontaneous emission coefficient B as a function of backside reflectance and grid reflectance for different device architectures. (a) B for LEDs with planar (P) and textured (T) mirrors with a haze factor of $h = 0.5$ emitting to air or into ZnSe (Z). (b) B for LEDs emitting into a reflective grid (Au) and lossy grid (Pd/Ge/Au alloy). (c) B for LEDs with different mirror architectures, with planar and textured mirrors. The average reflectivities of different grid and mirror structures from different mirror an grid designs from the main text are indicated with dotted lines.

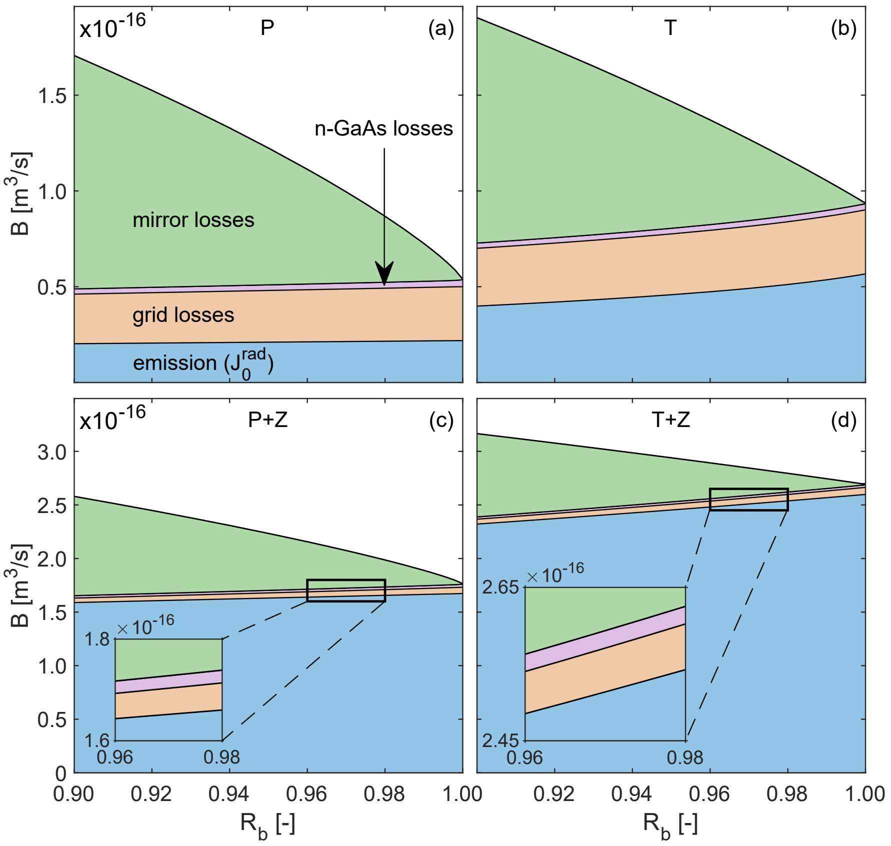

Figure S4: Trends in B as a function of backside reflectance broken down into separate loss and emission channels for devices with different light extraction schemes and a grid coverage of $g_c = 0.1$. (a) with a planar backside mirror and a lossy PD/Ge/Au grid (b) with a textured mirror with $h = 0.5$ and a lossy PD/Ge/Au grid (c) with a planar mirror, ZnSe encapsulation and a Au grid, and (d) with a textured mirror with $h = 0.5$, ZnSe encapsulation and a Au grid. The insets on the bottom row highlight the grid and n-GaAs loss channels

4 Comparison between TMM-RT and FED simulation

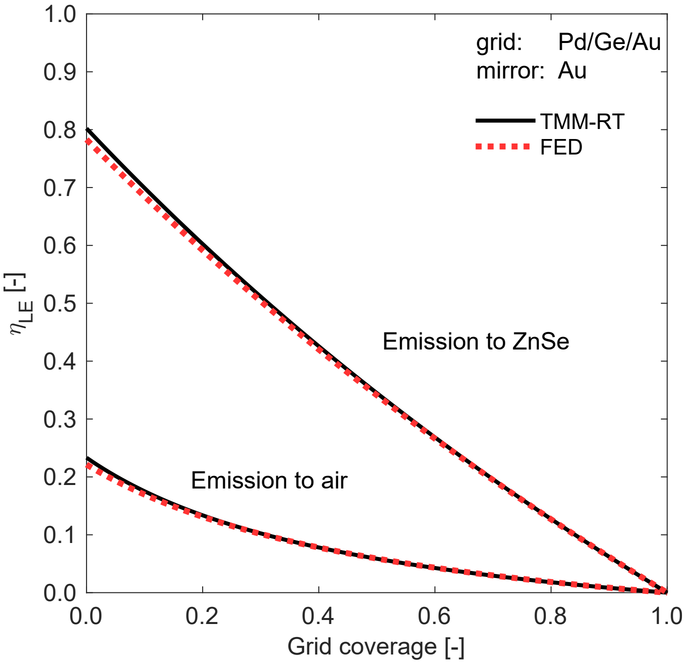

Figure S5: Comparison between η_{LE} as a function of grid coverage from TMM-RT simulation and from 1D fluctuational electrodynamics (FED) simulation with the same model as in previous work.[1] The device architecture considered has a Pd/Ge/Au alloy grid and a Au mirror, similar to Fig. 5b in the main text. Only planar structures are considered, since textured surfaces are not supported in the FED framework used here. The comparison shows good agreement between both methods. Note that the FED simulation is also 1D, and therefore the trends are calculated using equation 13 from the main text, in the same way η_{LE} is calculated when the TMM-RT method is used.

5 Derivation of equivalence of frameworks using total radiative current and *net* radiative current

In this derivation and in the main text, we have used η_{int} for the internal radiative efficiency and η_{ext} for the external radiative efficiency. In research related to LEDs, these quantities are generally reffered to as the internal quantum efficiency (IQE) and external quantum efficiency (EQE), respectively. They relate to efficiencies with which charge carriers generate photons. This is notably different from the IQE and EQE used in PV literature, where they refer to the opposite process, i.e. how efficiently photons are absorbed. We have opted to use η_{int} and η_{ext} as nomenclature since they do not have a double meaning between the two research communities.

For the comparison between the framework presented in the main text and the framework used in Steiner et al.,[9] which is often referred to in PV literature, we first define the relevant quantities. In the model by Steiner er al., the radiative recombination rate U_{rad} consists of the radiative recombination events ending up as (a) photons escaping into ambient, (b) photons reabsorbed by the active region (a process also known as photon recycling), and (c) photons parasitically absorbed without restoring the electron-hole pair. The probabilities of processes (a) and (b) are P_{esc} and P_{abs}, respectively. For

the purposes of this derivation, we can also define a probability for parasitic absorption, which is axiomatically given by $P_{par} = 1 - P_{esc} - P_{abs}$.
Steiner et al.[9] define η_{int} as

$$\eta^s_{int} = \frac{U_{rad}}{U_{rad} + U_{nr}}, \tag{13}$$

where U_{rad} and U_{nr} refer to the radiative and nonradiative recombination rate, respectively. The superscript s is the clarify this is the definition by Steiner et al. Since U_{rad} includes photons that are recycled and therefore restore the electron-hole pair for another recombination event, η^s_{ext} depends on η^s_{int}, P_{esc} and P_{abs} as

$$\eta^s_{ext} = \frac{\eta^s_{int} P_{esc}}{1 - \eta^s_{int} P_{abs}}. \tag{14}$$

The light extraction efficiency η_{LE} is not typically used in PV literature. In the Steiner framework, it has an explicit dependence on η^s_{int}, since

$$\eta_{LE} = \frac{\eta^s_{int}}{\eta^s_{ext}} = \frac{P_{esc}}{1 - \eta^s_{int} P_{abs}}. \tag{15}$$

In the framework presented in the main text, η_{int} is instead defined based on the *net* radiative recombination rate $U_{rad,net}$, given by

$$U_{rad,net} = (P_{esc} + P_{par})U_{rad} = (1 - P_{abs})U_{rad}. \tag{16}$$

The term net radiative recombination rate indicates that photons that are recycled are not included in $U_{rad,net}$. The alternative η_{int} is then given by

$$\eta_{int} = \frac{U_{rad,net}}{U_{rad,net} + U_{nr}}. \tag{17}$$

In the current framework, η_{LE} is based on the relevant net radiative recombination rates, resulting in

$$\eta_{LE} = \frac{P_{esc} U_{rad}}{U_{rad,net}} = \frac{P_{esc} U_{rad}}{(P_{esc} + P_{par})U_{rad}} = \frac{P_{esc}}{(1 - P_{abs})}. \tag{18}$$

To show the equivalence of both frameworks, it can be shown that $\eta_{ext} = \eta^s_{ext}$ as follows

$$\begin{aligned}
\eta_{ext} = \eta_{int}\eta_{LE} &= \frac{(1 - P_{abs})U_{rad}}{(1 - P_{abs})U_{rad} + U_{nr}} \frac{P_{esc}}{1 - P_{abs}} = \frac{P_{esc} U_{rad}}{(1 - P_{abs})U_{rad} + U_{nr}} \\
&= \frac{P_{esc} U_{rad}}{(1 - P_{abs})U_{rad} + U_{nr}} = \frac{P_{esc} U_{rad}}{U_{rad} + U_{nr} - P_{abs} U_{rad}} = \frac{\frac{U_{rad}}{U_{rad}+U_{nr}} P_{esc}}{\frac{U_{rad}+U_{nr}-P_{abs}U_{rad}}{U_{rad}+U_{nr}}} \\
&= \frac{\frac{U_{rad}}{U_{rad}+U_{nr}} P_{esc}}{\frac{U_{rad}+U_{nr}}{U_{rad}+U_{nr}} - \frac{U_{rad}}{U_{rad}+U_{nr}} P_{abs}} = \frac{\eta^s_{int} P_{esc}}{1 - \eta^s_{int} P_{abs}} = \eta^s_{ext}.
\end{aligned} \tag{19}$$

6 Analyses of additional structures

In figure S6, the same analyses is performed as for figure 4 in the main text, now using a 2000 nm active layer, which is more typical for III-V thin film PV cells. We note that the trends are not strongly affected, compared to a structure with a 300 nm active layer. However, there is a large

difference in $J_{0,int}^{rad}$, which for a 2000 nm active layer is nearly two times larger in the cases without a dome, and roughly 2.5 times larger with a dome. An increase in internally generated radiative current is expected when the active layer thickness is increased. The effect of texturization on the increase in emission is also reduced, which is similarly expected, since the behavior of the device becomes more dominated by the absorption and emission in the active layer as it increases in thickness.[10] This can be understood by considering that in an infinitely thick cell, a scattering mirror does not impact performance, as the optical flux will reach the exact same steady state (balance between absorption and emission in the active layer), regardless of loss processes or redistribution of light intensity at the backside. The radiance after a single pass will tend to this value, and the thicker the active layer, the closer the radiance will be to that value. Note that the assumption that the area with grid and the area without grid can be treated separately is equally valid here, since the average lateral distance traveled using a 2000 nm active layer is similar to that in a 300 nm active layer, as a result of the higher reabsorption rate of photons in thick active layers.[10]

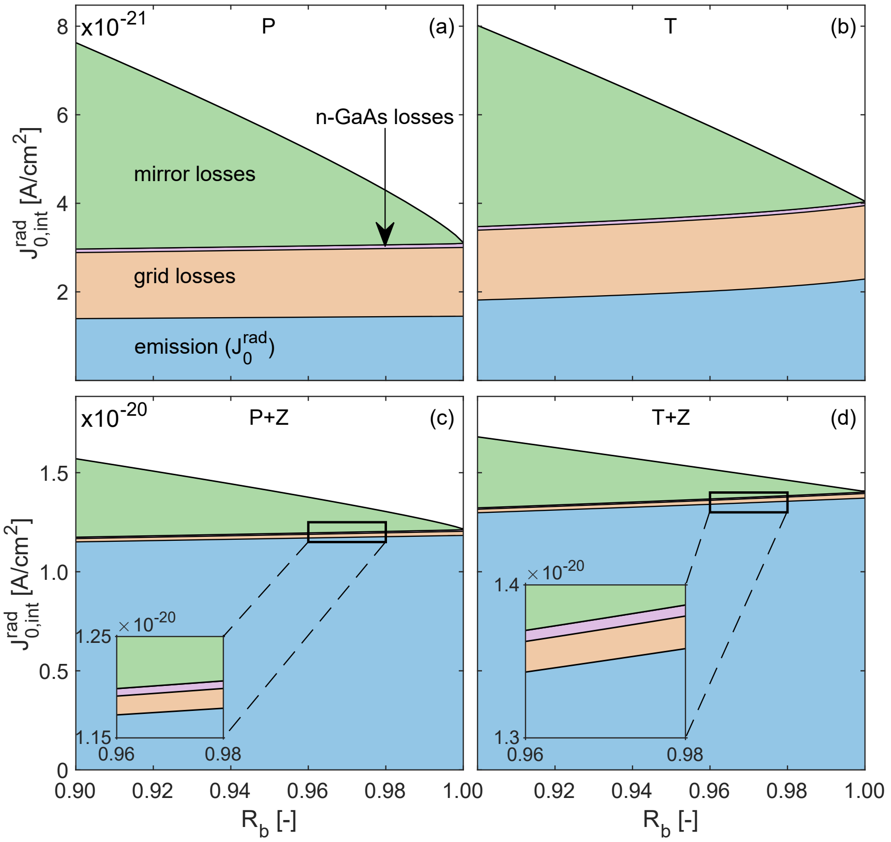

Figure S6: Trends in $J_{0,int}^{rad}$ as a function of backside reflectance broken down into separate loss and emission channels for devices with different light extraction schemes and a grid coverage of $g_c = 0.1$. This device differs from the standard device in the main text, now having a 2000 nm active layer, more typical for thin-film III-V PV cells. (a) with a planar backside mirror and a lossy PD/Ge/Au grid (b) with a textured mirror with $h = 0.5$ and a lossy PD/Ge/Au grid (c) with a planar mirror, ZnSe encapsulation and a Au grid, and (d) with a textured mirror with $h = 0.5$, ZnSe encapsulation and a Au grid. The insets on the bottom row highlight the grid and n-GaAs loss channels.

Similar to in figure S6, in figure S7, the same analyses is performed as for figure 4 in the main text, but now using 200 nm contacts (and a 300 nm active layer, as in the main text). This is a

tenfold increase in thickness, aimed at showing the effect of more absorptive contact layers. Since the backside reflectivity is parametrized, this shows up only as an increase the losses in the topside n-GaAs contact layer. However, even for 200 nm contact layers, this loss is far smaller than the losses of an absorptive Pd/Ge/Au grid (top row). When reflective Au grids are used, and the light extraction efficiency is increased using ZnSe encapsulation, the n-GaAs contact becomes the dominant loss mechanism at high mirror reflectivities. This indicates that in devices with low losses and high extraction efficiencies, the contact layer losses can becomes significant. These losses can be alleviated by utilizing thinner contact such as in the main text. For the n-GaAs contact layer, utilizing higher doping levels than in this study ($6.7 \cdot 10^{18}$ cm^{-3} for n-GaAs), would increase the transparency to the internal emission, as the bandfilling effect shifts the band edge to higher energies. Alternatively, different materials can be explored, such as AlGaAs, which is intrinsically transparent to the emission of GaAs.

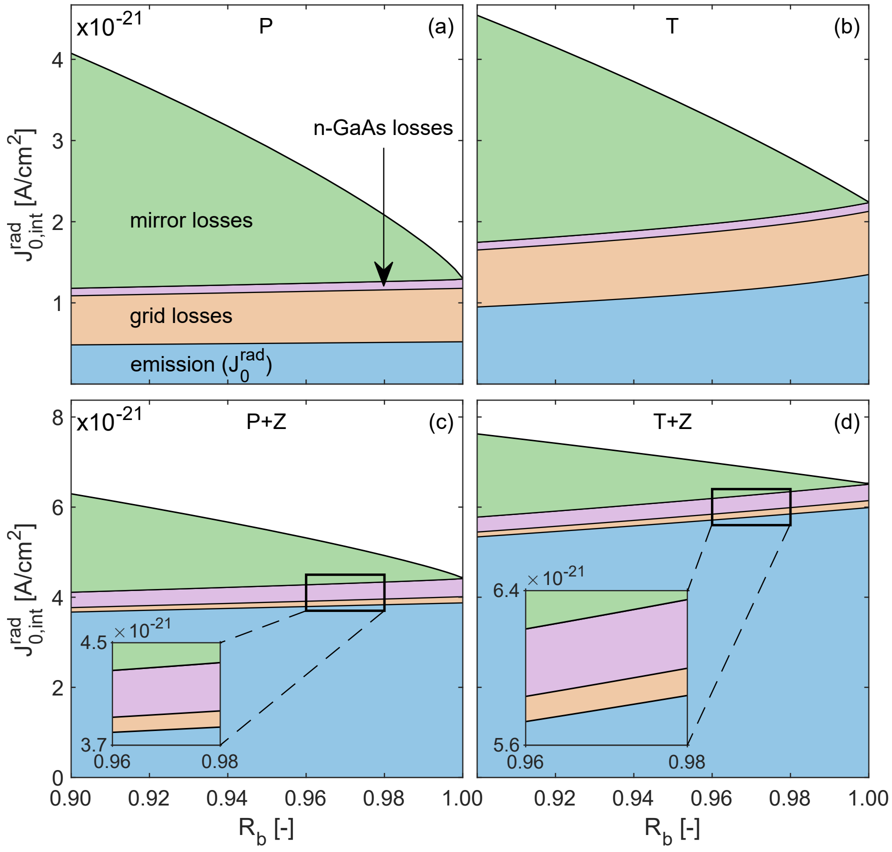

Figure S7: Trends in $J_{0,int}^{rad}$ as a function of backside reflectance broken down into separate loss and emission channels for devices with different light extraction schemes and a grid coverage of $g_c = 0.1$. This device differs from the standard device in the main text, now having a 200 nm contact layers. (a) with a planar backside mirror and a lossy PD/Ge/Au grid (b) with a textured mirror with $h = 0.5$ and a lossy PD/Ge/Au grid (c) with a planar mirror, ZnSe encapsulation and a Au grid, and (d) with a textured mirror with $h = 0.5$, ZnSe encapsulation and a Au grid. The insets on the bottom row highlight the grid and n-GaAs loss channels.

7 Photovoltaic V_{oc} analyses

In figure S8, ΔV_{oc} is plotted using

$$\Delta V_{oc} = V_{db} - V_{oc} = -\frac{kT}{q}\ln(\eta_{ext}), \tag{20}$$

where V_{db} is the open circuit voltage in the radiative limit, or detailed-balance voltage. ΔV_{oc} is a metric catered to PV cells. We assume here that $\eta_{int} = 1$, such that $\eta_{ext} = \eta_{LE}$. This allows us to directly use the data from figure 5 in the main text to calculate ΔV_{oc}. Note that V_{db} is different for the different architectures, since it depends on the external quantum efficiency for absorption (EQE), which is device specific. Particularly in textured devices, this quantity is found to be smaller than in planar devices.[10] As such, even though the V_{oc} penalty for textured structures is lower, this does not imply an increase in V_{oc} directly.

As a result of the logarithmic dependence of ΔV_{oc} on η_{LE}, the non-linearity in ΔV_{oc} is less pronounced. Note that PV cells typically do not employ extraction media. Nevertheless, their trends are reported here for completeness. Comparing figure S8a to S8b, where a lossy Au mirror is employed, at 10% grid coverage, ΔV_{oc} is about 4 mV smaller when emitting to air and 2 mV smaller when emitting into ZnSe when using an Au grid, compared to a Pd/Ge/Au grid, regardless of the texturization of the backside mirror. In figures S8c and S8d, where the mirror is less lossy, the effect on η_{LE} is more pronounced as can be observed in figure 5 of the main text. By extension, the effect on ΔV_{oc} is also larger. Here, again about 2 mV difference is found when emitting into ZnSe between the different grid architectures, similar to the case with a Au mirror. However, more notable is that without an extraction medium, the difference is about 12 mV for planar cells and 8 mV for textured cells with a haze factor of 0.5. This difference is significant in high performance III-V cells, and strongly impacts cell efficiency, such as shown for example by Miller et al.[11] In summary, in certain PV cell architectures, a strongly absorbing grid impacts cell performance through its effect on the V_{oc}.

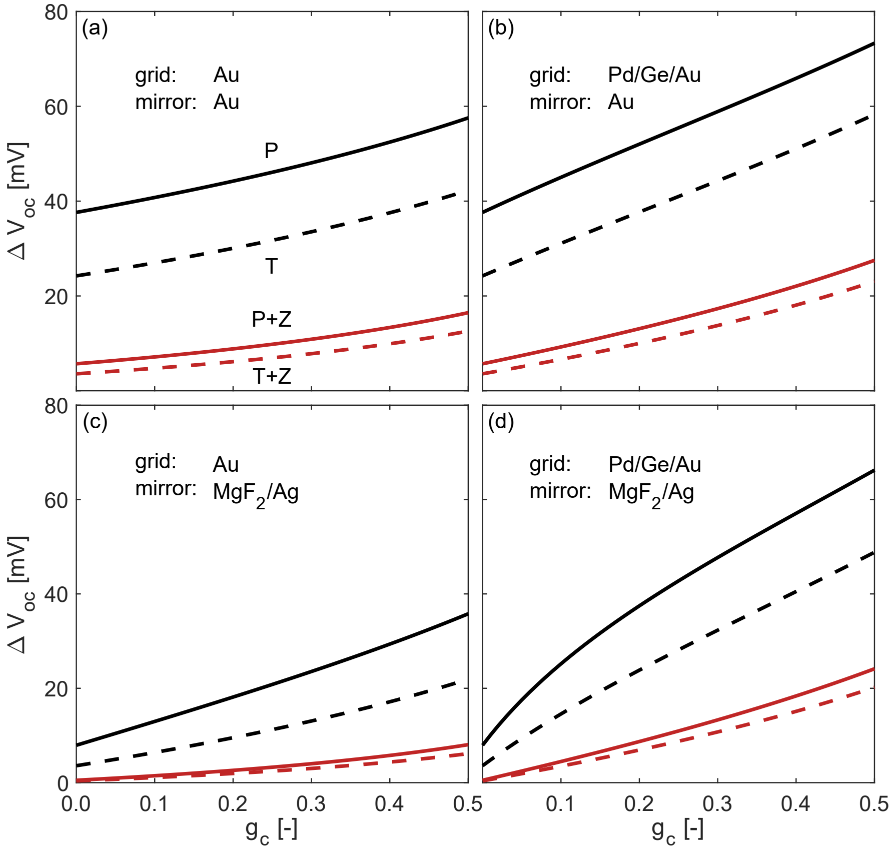

Figure S8: Trends in $J_{0,int}^{rad}$ as a function of backside reflectance broken down into separate loss and emission channels for devices with different light extraction schemes and a grid coverage of $g_c = 0.1$. This device differs from the standard device in the main text, now having a 2000 nm active layer, more typical for thin-film III-V PV cells. (a) with a planar backside mirror and a lossy PD/Ge/Au grid (b) with a textured mirror with $h = 0.5$ and a lossy PD/Ge/Au grid (c) with a planar mirror, ZnSe encapsulation and a Au grid, and (d) with a textured mirror with $h = 0.5$, ZnSe encapsulation and a Au grid. The insets on the bottom row highlight the grid and n-GaAs loss channels.

References

[1] van Gastel, J.; Kivisaari, P.; Oksanen, J.; Vlieg, E.; Schermer, J. J. Optical analyses of lossy near-field thermophotonic devices with planar and scattering mirrors. *Solar Energy Materials and Solar Cells* **2025**, *282*, 113331.

[2] Chuang, S. *Physics of Photonic Devices*; Wiley: Hoboken, new Jersey, 2009.

[3] Wurfel, P. The chemical potential of radiation. *Journal of Physics C: Solid State Physics* **1982**, *15*, 3967–3985.

[4] Centurioni, E. Generalized matrix method for calculation of internal light energy flux in mixed coherent and incoherent multilayers. *Applied Optics* **2005**, *44*, 7532–7539.

[5] SOPRA Database. *http://www.sspectra.com/sopra.html*

[6] Jiang, Y.; Pillai, S.; Green, M. A. Realistic Silver Optical Constants for Plasmonics. *Scientific Reports* **2016**, *6*, 30605.

[7] Johnson, P. B.; Christry, R. W. Optical constants of the noble metals. *Physical Review B* **1972**, *6*, 4370–4379.

[8] Casey, H. C.; Stern, F. Concentration-dependent absorption and spontaneous emission of heavily doped GaAs. *Journal of Applied Physics* **1976**, *47*, 631–643.

[9] Steiner, M. A.; Geisz, J. F.; García, I.; Friedman, D. J.; Duda, A.; Kurtz, S. R. Optical enhancement of the open-circuit voltage in high quality GaAs solar cells. *Journal of Applied Physics* **2013**, *113*, 123109.

[10] van Eerden, M.; van Gastel, J.; Bauhuis, G. J.; Vlieg, E.; Schermer, J. J. Comprehensive analysis of photon dynamics in thin-film GaAs solar cells with planar and textured rear mirrors. *Solar Energy Materials and Solar Cells* **2022**, *244*, 111708.

[11] Miller, O. D.; Yablonovitch, E.; Kurtz, S. R. Strong internal and external luminescence as solar cells approach the Shockley-Queisser limit. *IEEE Journal of Photovoltaics* **2012**, *2*, 303–311.
